# No causal association between plasma cystatin C and cardiovascular diseases: Mendelian randomization analyses in UK biobank

**DOI:** 10.3389/fmed.2023.1191675

**Published:** 2023-08-17

**Authors:** Jingjing Tu, Ying Xu, Xu Guo, Jiayu Zhang, Duo Xu, Liyuan Han, Yue Wang, Boya Zhang, Hongpeng Sun

**Affiliations:** ^1^Department of Rehabilitation Medicine, Ningbo No.2 Hospital, Ningbo, China; ^2^School of Public Health, Medical College of Soochow University, Suzhou, China; ^3^Department of Global Health, Ningbo Institute of Life and Health Industry, University of Chinese Academy of Sciences, Ningbo, Zhejiang, China

**Keywords:** plasma cystatin C, cardiovascular disease, stroke, myocardial infarction, Mendelian randomization, genetic risk

## Abstract

**Background:**

We aimed to determine whether the plasma cystatin C is a causal risk factor for cardiovascular events, stroke, myocardial infarction (MI), and cardiovascular disease (CVD) mortality by conducting Mendelian randomization (MR) designs.

**Methods:**

Our study included 277,057 individuals free of CVDs or cancer at baseline in the UK Biobank. The genetic scores of plasma cystatin C comprising 67 single-nucleotide polymorphisms were calculated on the basis of data from a large genome-wide association study. By stratifying the genetic score, we conducted cox regression to assess the relationship between plasma cystatin C and CVDs. In this study, linear MR analysis was used to estimate the causal association between plasma cystatin C and CVDs.

**Results:**

Observational analyses showed that plasma cystatin C concentrations were associated with the risk of CVDs [hazard ratios (HR) per standard deviation (SD) 1.09, 95% confidence interval (CI); 1.07–1.10] and CVD mortality (1.14, 1.11–1.17). Among CVDs, plasma cystatin C were associated with stroke (1.10, 1.08–1.11) and MI (1.08, 1.07–1.10). Linear MR analysis did not provide evidence of a causal association between plasma cystatin C and the risk of CVDs [odds ratio (OR) per SD 0.96, 95% CI;0.90–1.03], stroke (0.96, 0.93–1.01), MI (0.97, 0.91–1.03), and CVD mortality (0.98, 0.96–1.01), with consistent estimates from sensitivity analyses.

**Conclusion:**

Observational findings indicated that higher plasma cystatin C is associated with a higher risk of CVDs; According to MR studies, there is no causal association between plasma cystatin C and the risk of CVDs and CVD mortality.

## 1. Introduction

Cystatin C is a non-glycosylated, low-molecular-weight protein. It belongs to the cystatin superfamily of cysteine protease inhibitors and primarily controls the activity of extracellular proteases (1, 2). In clinical settings, cystatin C is often used as a surrogate for serum creatinine to assess renal function because blood cystatin C concentrations are not affected by age, sex, or smoking habits (3, 4).

Previous prospective studies have reported that serum cystatin C concentrations are associated with the risk of coronary heart disease (CHD), myocardial infarction (MI), heart failure, and secondary cardiovascular events (5–7). In addition, cystatin C is closely associated with cardiovascular disease (CVD) risk factors, such as hypertension, aging, and diabetes (8). A prospective cohort study showed that for every 0.2 mg/L increase in the plasma cystatin C, the incidence of hypertension increased by 15% (9). In contrast, another study reported that plasma cystatin C concentrations were not associated with CVDs (10). In addition, a clinical study has found serum cystatin C is a reliable indicator of renal function in patients with systemic lupus erythematosus. However, it is not independently associated with cardiovascular risk factors or subclinical atherosclerosis (11).

At present, the causal relationship between Cystatin C concentration and CVDs was still unclear. The findings of traditional observational studies are prone to residual confounding effects and reverse causality. In addition, some factors in the multivariate analyses, such as thyroid function, remain uncorrected (12). Mendelian randomization (MR) studies are considered naturally occurring randomized clinical trials because parental alleles are randomly assigned to individuals (13). Therefore, the association between the genes and outcomes is not affected by confounding factors such as environmental and behavioral factors after birth. Hence, genetic variation has been used as an instrumental variable to estimate the causal association between plasma cystatin C concentrations and CVDs.

In this large-scale prospective study using UK Biobank genetic data, we first assessed the associations between plasma cystatin C concentrations and the risk of CVDs using Cox regression in observational analysis. Next, the two-stage least-squares method was used to examine genetic evidence for the associations between plasma cystatin C concentrations and the incidence of CVDs. Finally, using genetic variants associated with plasma cystatin C previously published in meta-analyses of genome-wide association studies, instrumental variable analysis was used to assess the causal association of plasma cystatin C with the risk of CVDs.

## 2. Materials and methods

### 2.1. Study cohort

The UK Biobank is a data repository from a prospective cohort study that was conducted at 22 assessment centers between 2006 and 2010 (14, 15). It contains more than 500,000 aged 40–69 participants in the genetic, body and health data.^1^ In the UK Biobank study, health information was collected through a touch screen questionnaire, interviews, and physical measurements. Blood samples were collected for genotyping and biomarker analysis. The study design and details of quality control have been published previously (16). The participants provided written informed consent, and ethical approval was obtained from the UK National Health Service’s National Research Ethics Service (ref 11/NW/0382).

In this study, participants with cancer and CVDs at baseline (*n* = 79,026) and those with missing values of plasma cystatin C concentration (*n* = 25,219) were excluded. For the genetic analysis, the genetic data from 277,057 unrelated individuals of European ancestry were retrieved from the UK Biobank and used in our analysis. Supplementary Figure S1 describes the inclusion and exclusion process for the study subjects.

### 2.2. Ascertainment of plasma cystatin C concentrations and outcomes

The UK Biobank has quantified the concentrations of various biochemical markers using the biological samples collected from all participants at baseline. The samples were collected from approximately 480,000 participants who were included via recruitment interviews, and approximately 18,000 samples were collected in repeated assessments. Plasma cystatin C concentrations (mg/L) were measured and the result is available on the UK Biobank website.^2^

The primary outcomes of our study were cardiovascular events (stroke, MI) and CVD mortality. The secondary outcomes included MI and stroke. Information on cardiovascular events and the time at which the events occurred is based on certified death records and cumulative medical records of hospital diagnoses. All CVD events were defined with 3-digit codes according to the International Classification of Diseases 10th Revision.^3^ EachCVD events was defined as follows: CVD mortality (I00–I99), stroke (I60–I64) and MI (I21–I23, I24.1, or I25.2).

### 2.3. Selection of SNPs and genetic risk score as instrumental variables

A previous study described the genotyping process and arrays used in the UK Biobank study (17). Sixty-seven single-nucleotide polymorphisms (SNPs) were selected, all of which were genome-wide significant variants (*p* < 5 × 10^−8^), discovered in a recent published genome-wide association analysis, for plasma cystatin C concentrations in the UK Biobank (18). Supplementary Table S1 presents information about selected SNPs. Depending on the number of risk alleles contained, individual SNPs were coded as 0 (no risk allele), 1 (one risk allele), and 2 (two risk alleles). The genetic risk score (GRS) was determined by calculating the weighted average of the number of individual alleles that are positively associated with the cystatin C concentration and then multiplying the average by the number of available variants (19). The effect size coefficient of each SNP was selected from the published genome-wide association analysis. The effect size coefficient of each SNP indicates that each additional effect allele of this genetic locus corresponds to the cystatin C effect size at the element level.

### 2.4. Statistical analysis

Baseline characteristics were described as the number (percentage) of categorical variables, mean (standard deviation) for symmetrical continuous variables, and median (interquartile range) for asymmetrical continuous variables. We used Cox proportional hazards models to estimate hazard ratios (HRs) for cardiovascular risk. The samples for plasma cystatin C concentration measurement were divided into five equal groups (<0.78 mg/L, 0.78–0.85 mg/L, 0.85–0.92 mg/L, 0.92–1.00 mg/L, >1.00 mg/L), each separated by one standard deviation (SD). Analyses were conducted using the following three models: (1) adjusted for age, sex, Townsend Deprivation Index (continuous), physical activity, smoking status, drinking status, annual household income (<£18,000, £18,000–£52,000, £52,000–£100,000, >£100,000), and employment (yes or no); (2) additionally adjusted for body mass index (continuous), high-density lipoprotein (HDL) cholesterol (continuous), and total cholesterol; and (3) additionally adjusted for the presence of diabetes (yes or no), hypertension (yes or no), and chronic kidney disease (yes or no).

For genetic analysis, we used Cox regression models to assess the relationship between cystatin C concentrations and cardiovascular events, adjusted for age, sex, and top 10 genetic principal components (20). The participants were divided into three groups according to their GRS quartiles: upper quartile (Q3; the group with the highest GRS), lower quartile (Q1; the group with the lowest GRS), and interquartile (Q2–Q3; the group with an intermediate GRS). We also performed Cox regression to assess the HR of each cardiovascular event per unit increase in GRS. To effectively control for confounding factors, we conducted 3 models and adjusted for traditional risk factors for CVDs as previously described (21, 22). Furthermore, the validity of genetic variations was assessed by examining the associations of potential confounders with the GRS, thus avoiding possible violations of the MR hypothesis (Supplementary Table S3).

For the linear MR analyses, the two-stage least-squares method was used to estimate the relationships between the genetically predicted cystatin C concentrations and cardiovascular risk. First, we performed linear regression to match the cystatin C concentration with the GRS, and then performed logistic regression models to assess the association between GRS and CVDs. Both steps were adjusted for age, sex, genotyping arrays, and the top 10 principal components.

In the sensitivity analysis, potentially invalid SNPs associated with confounding factors (e.g., BMI, cholesterol, creatinine concentrations) and/or indications of known pleiotropic effects based on selected genotype-to-phenotype catalogs (i.e., GWAS-Catalog and PhenoScanner) were excluded, and another set of instrumental variables (Supplementary Table S2) was generated. Inverse-variance weighting (IVW) (23), weighted median (24), and MR-Egger (25) were used to assess the association of cystatin C concentrations with CVDs to assess the robustness of our results. Then, the MR-Egger intercept and MR-pleiotropy residual sum and outlier (MR-PRESSO) global test were used to identify the potential horizontal pleiotropic effects of the SNPs (26). Cochran’s Q test was used to assess the heterogeneity between causal estimates from different genetic variants, which can help detect pleiotropy (27).

To analyze observational associations, MR analyses were performed using SAS version 9.4 (SAS Institute Inc., Cary, NC) and R4.1.1 (R Development Core Team, Vienna, Austria). Sensitivity analyses were performed using the R package, two-sample MR^4^ (28), and MR-PRESSO^5^ (26). All *p* values for the tests were bilateral, and *p* values <0.05 were considered as statistically significant.

### 2.5. Ethics approval

UK Biobank has received ethics approval from the National Health Service National Research Ethics Service (ref 11/NW/0382). A statement confirming that all methods were carried out in accordance with relevant guidelines and regulations.

## 3. Results

### 3.1. Baseline characteristics

The baseline characteristics of the UK Biobank individuals are presented in Supplementary Table S4. A total of 372,882 individuals [mean age (SD): 56.92 years (8.07); 45.05% of males] were included in the observational analysis and 277,057 individuals [mean age (SD): 56.90 years (8.08); 44.59% of males] were included in the MR analysis. The mean plasma cystatin C concentration was 0.90 mg/L (SD 0.16 mg/L).

Table 1 shows the baseline characteristics of the individual in the MR analysis according to the GRS quartiles. Participants with a higher GRS were more likely to have a higher body mass index, plasma cystatin C concentration, and creatinine concentration and lower HDL, LDL, and serum total cholesterol concentrations. They also had higher rates of hypertension and chronic kidney disease than those with a lower GRS.

**Table 1:** Baseline characteristics of participants stratified by the quartiles of genetic risk score.

Quartiles categories of genetic risk score				
Characteristics	Lowest GRS	Intermediate GRS	Highest GRS	*p*
	(< 25%)	(25 to 75%)	(> 75%)	
N	69,064	137,851	70,142	
Age (years)	56.92 (8.10)	56.88 (8.09)	56.92 (8.12)	0.40
Sex, male (%)	44.63	44.51	44.69	0.65
BMI (kg/m^2^)	27.14 (4.61)	27.21 (4.67)	27.27 (4.69)	<0.01
Townsend deprivation index	−1.56 (2.93)	−1.56 (2.93)	−1.56 (2.93)	0.94
Current drinkers (%)	93.92	93.88	93.96	0.81
Current smokers (%)	9.35	9.42	9.17	0.12
Employment (%)	59.79	59.49	59.26	0.08
Physical activity (min/week)				0.59
<250	55.70	55.58	55.85	
250–550	23.78	23.94	23.82	
>550	20.52	20.49	20.33	
Income				<0.01
<18,000£	20.18	20.36	20.68	
18,000£-52,000£	51.99	51.74	51.47	
52,000£-100,000£	21.96	21.78	21.78	
>100,000£	5.88	6.11	6.06	
HDL, mmol/L	1.46 (0.37)	1.46 (0.37)	1.45 (0.37)	0.04
LDL, mmol/L	3.59 (0.87)	3.58 (0.86)	3.56 (0.86)	<0.01
Cholesterol mmol/L	5.75 (1.14)	5.73 (1.13)	5.70 (1.13)	<0.01
Triglycerides, mmol/L	1.72 (1.01)	1.72 (1.01)	1.72 (0.99)	0.04
Glucose, mmol/L	5.08 (1.10)	5.08 (1.13)	5.08 (1.10)	0.52
Creatinine, umol/L	71.40 (16.99)	71.84 (15.78)	72.47 (17.06)	<0.01
Cystatin C, mg/L	0.88 (0.16)	0.90 (0.16)	0.92 (0.17)	<0.01
Diabetes (%)	4.08	4.11	4.18	0.62
Hypertension (%)	37.82	38.07	39.03	<0.01
CKD (%)	2.25	2.46	2.51	<0.01

CKD: chronic kidney disease. Values were expressed as mean ± standard deviation, or n (%). ^*^p < 0.05.

### 3.2. Observational estimates of the association between plasma cystatin C and CVDs

**Figure 1:**
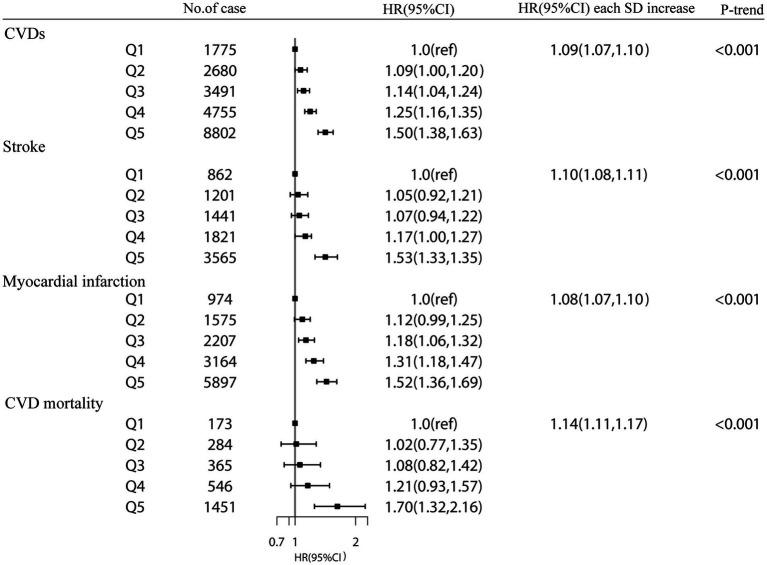
The association between plasma cystatin C and CVD events and CVD mortality in the UK Biobank study. Results were adjusted for age, sex, Townsend Deprivation Index (continuous), household income (<£18 000, £18 000-£52000, £52000-£100000, or >£100000), physical activity (<250 min/week, 250-550 min/week, >550 min/week), smoking status (never, former, current), drinking status (never, former, current), employment (no, yes), BMI (continuous), HDL cholesterol (continuous), LDL cholesterol (continuous), total cholesterol, diabetes (yes or no), hypertension (yes or no) and chronic kidney disease (yes or no). Hazard Ratios (HRs) at each category Q2-Q5 (compared with Q1) and per 1-standard deviation (SD) of each plasma cystatin C, estimated from Cox regression models. P value for trend was calculated as the trend per group.

During a mean follow-up duration of 9.94 years, 21,503 CVD events (8,890 strokes, 13,817 MIs, and 2,819 CVD mortalities) occurred. Higher cystatin C were related to a higher risk of CVDs. The HR for each SD of plasma cystatin C was 1.14 (95% CI = 1.13–1.15) (model 1). The HRs of plasma cystatin C in groups 2, 3, 4, and 5 compared with group 1 (the lowest group) were 1.07 (0.98, 1.77), 1.13 (1.04, 1.24), 1.28 (1.18, 1.39), and 1.74 (1.60, 1.88), respectively (Supplementary Table S5). In the multivariate model, HR per SD was 1.09 (95% CI = 1.07–1.10; p for trend <0.001) in model 3 (Figure 1).

In addition, higher plasma cystatin C concentrations were associated with a higher risk of stroke. Compared with group 1, the HRs of groups 2, 3, 4, and 5 were 1.05 (0.92, 1.21), 1.07 (0.94, 1.22), 1.17 (1.00, 1.27), and 1.50 (1.33, 1.70), respectively. The hazard ratio for each SD of plasma cystatin C was 1.10 (1.08, 1.11) (model 3). For each SD increase in plasma cystatin C, the hazard ratio for MI was 1.08 (1.07, 1.10) (Figure 1). For each SD increase in plasma cystatin C, the hazard ratio for CVD mortality was 1.14 (1.11, 1.17) (model 3).

**Figure 2:**
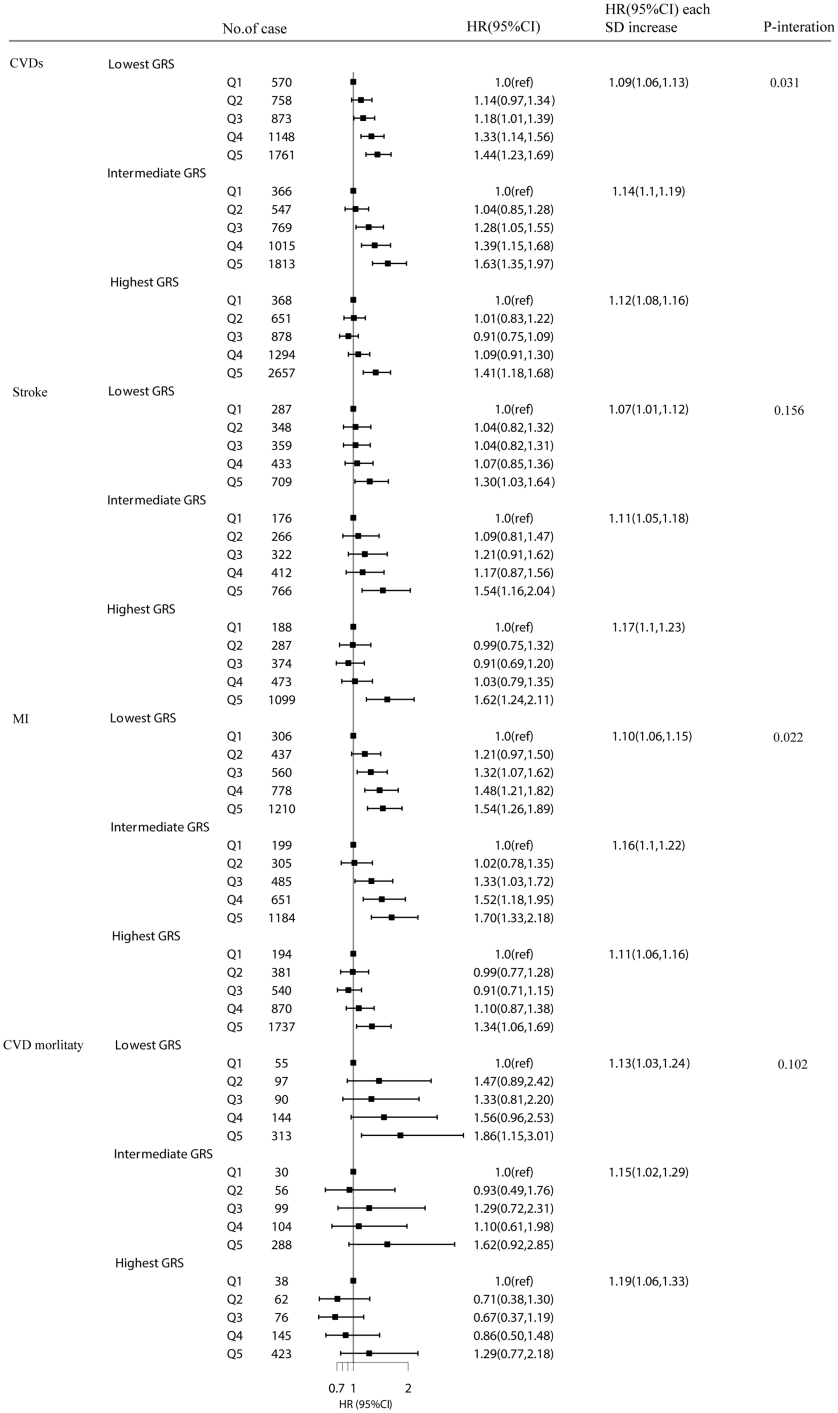
The association between plasma cystatin C with CVD events stratified by Cystatin C–GRS. Model were adjusted for age, sex, TDI (continuous), household income (<£18 000, £18 000-£52000, £52000-£100000, or >£100000), physical activity (<250 min/week, 250-550 min/week, >550 min/week), smoking status (never, former, current), drinking status (never, former, current), employment (no, yes), BMI (continuous), HDL cholesterol (continuous), LDL cholesterol (continuous) and total cholesterol, diabetes (yes or no), hypertension (yes or no) and chronic kidney disease (yes or no).

A stratified analysis based on cystatin C–GRS were performed to assess whether plasma cystatin C is related to the risk of CVDs. There was a significant association between plasma cystatin C and cystatin C–GRS, which had an effect on the risk of total CVDs (p for interaction = 0.031) and MI (p for interaction = 0.022). The HR of the risk of CVDs associated with each SD of plasma cystatin C was 1.09 (1.06–1.13) in the lowest GRS group, 1.14 (1.11–1.19) in the medium GRS group, and 1.12 (1.08–1.16) in the highest GRS group (Figure 2).

### 3.3. Association between cystatin C–GRS of plasma cystatin C and CVDs

To assess whether a higher cystatin C–GRS is related to a lower HR for CVDs, we investigated the association between cystatin C–GRS and CVDs using Cox proportional hazards models (Table 2). For CVDs, compared with the HR for the lowest quartile of cystatin C–GRS, participants with the highest quartile of cystatin C–GRS was 0.97 (0.92, 1.03; *p* = 0.31) in model 1. These associations did not change after further adjustment for other lifestyle behavioral, and biochemical factors in models 2 and 3. Our results showed that each SD increment of cystatin C–GRS was negatively associated with CVDs (HR 0.99; 95% CI = 0. 96–1.01), stroke (HR 0.98; 95% CI = 0. 94–1.01), MI (HR 0.98; 95% CI = 0. 96–1.01), and CVD mortality (HR 0.96; 95% CI = 0. 90–1.02) (model 3).

**Table 2:** Associations between genetic risk score and CVD events and CVD mortality in UK Biobank study.

Genetic risk score							
	Lowest GRS	Intermediate GRS	*p*	Highest GRS	*p*	P for trend	*p*
		HR (95%CI)		HR (95%CI)		HR (95%CI)	
CVDs							
model 1	1.0 (ref)	0.99 (0.94, 1.06)	0.90	0.97 (0.92, 1.03)	0.31	1.00 (0.97, 1.02)	0.78
model 2	1.0 (ref)	0.99 (0.93, 1.05)	0.95	0.90 (0.90, 1.01)	0.09	0.99 (0.97, 1.02)	0.32
model 3	1.0 (ref)	0.99 (0.93, 1.05)	0.73	0.95 (0.90, 1.01)	0.08	0.99 (0.96, 1.01)	0.28
Stroke							
model 1	1.0 (ref)	1.05 (0.95, 1.15)	0.37	0.95 (0.86, 1.04)	0.24	0.99 (0.95, 1.02)	0.42
model 2	1.0 (ref)	1.04 (0.94, 1.15)	0.44	0.93 (0.85, 1.02)	0.12	0.98 (0.94, 1.01)	0.22
model 3	1.0 (ref)	1.04 (0.94, 1.15)	0.44	0.93 (0.85, 1.02)	0.10	0.98 (0.94, 1.01)	0.19
MI							
model 1	1.0 (ref)	0.97 (0.90, 1.05)	0.42	0.96 (0.90, 1.03)	0.30	0.99 (0.97, 1.02)	0.66
model 2	1.0 (ref)	0.96 (0.90, 1.04)	0.34	0.94 (0.90, 1.01)	0.10	0.98 (0.96, 1.01)	0.29
model 3	1.0 (ref)	0.96 (0.89, 1.04)	0.32	0.94 (0.88, 1.01)	0.09	0.98 (0.96, 1.01)	0.27
CVD mortality							
model 1	1.0 (ref)	0.99 (0.83, 1.17)	0.89	0.89 (0.76, 1.05)	0.16	0.96 (0.90, 1.02)	0.21
model 2	1.0 (ref)	0.98 (0.82, 1.17)	0.84	0.88 (0.75, 1.05)	0.13	0.96 (0.90, 1.02)	0.18
model 3	1.0 (ref)	0.99 (0.83, 1.17)	0.87	0.88 (0.75, 1.04)	0.13	0.96 (0.90, 1.02)	0.19

CVD: cardiovascular disease; Myocardial infarction: MI; GRS: genetic risk score; OR: odds ratio. Mode 1:adjusted for age, sex, Townsend Deprivation Index (continuous), household income (<£18,000, £18,000-£52,000,£52,000–£100,000, or > £100,000), physical activity (<250 min/week, 250–550 min/week, >550 min/week), smoking status (never, former, current), drinking status (never, former, current), employment (no, yes). Model 2: mode 1 + BMI (continuous), HDL cholesterol (continuous), LDL cholesterol (continuous) and total cholesterol. Model 3: model 2 + diabetes (yes or no), hypertension (yes or no) and chronic kidney disease (yes or no).

^*^p < 0.05.

### 3.4. Linear MR analyses of plasma cystatin C with CVDs

**Figure 3:**
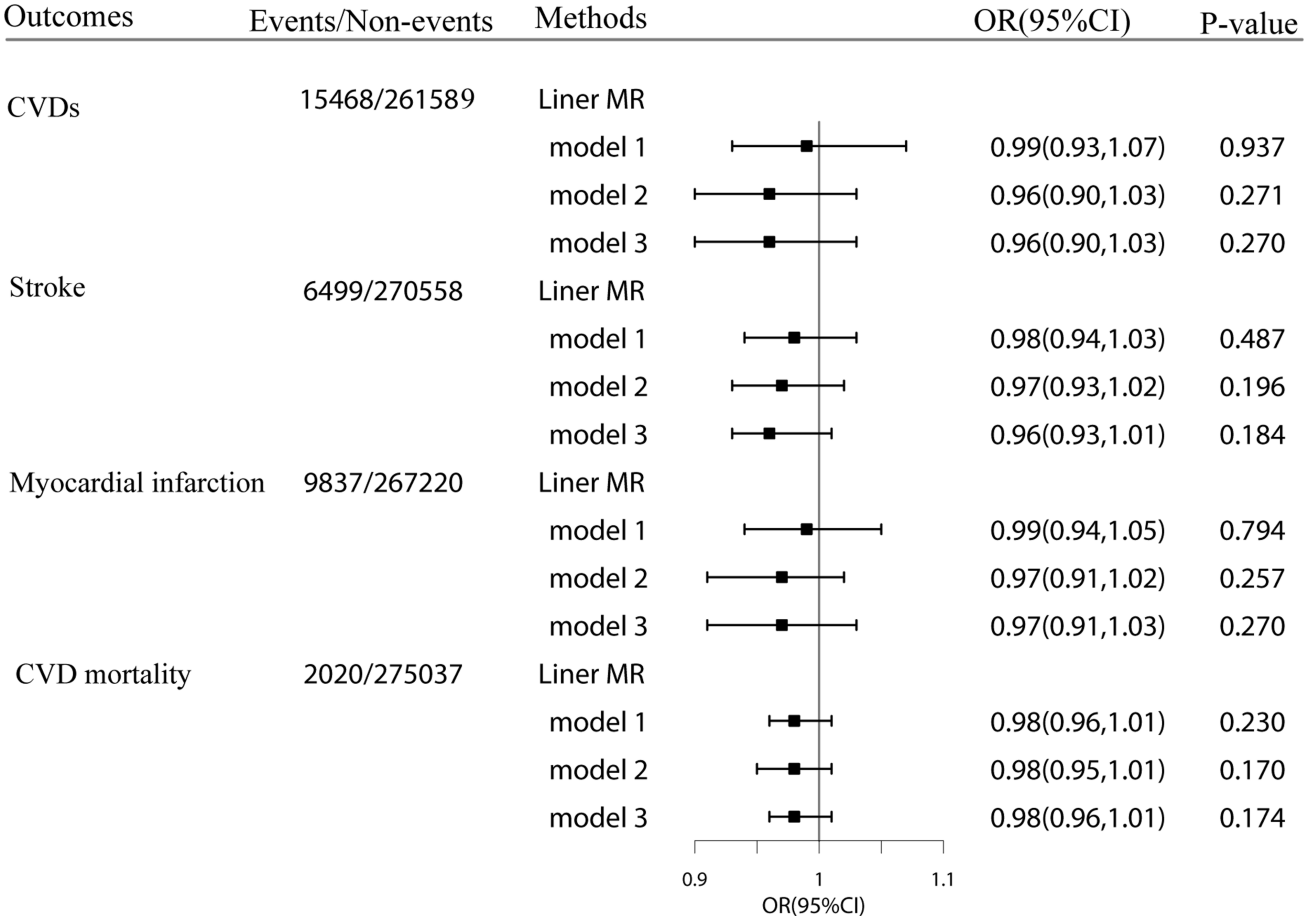
Linear Mendelian randomization estimates for the associations of genetically predicted plasma cystatin C with CVD events and CVD mortality in the UK Biobank. Odds ratios were estimated by two-stage least squares regression method. Model1:adjusted for age, sex, Townsend Deprivation Index (continuous), household income (<£18 000, £18 000-£52000, £52000-£100000, or >£100000), physical activity (<250 min/week, 250-550 min/week, >550 min/week), smoking status (never, former, current), drinking status (never, former, current), employment (no, yes). Model2: model1 + BMI (continuous), HDL cholesterol (continuous), LDL cholesterol (continuous) and total cholesterol. Model3: model2 + diabetes (yes or no), hypertension (yes or no) and chronic kidney disease (yes or no).

Our linear MR analyses showed no causal role of genetically predicted plasma cystatin C concentrations in CVDs (odds ratio [OR]: 0.96 per SD increment of plasma cystatin C; 95% CI = 0.90–1.03; *p* = 0.27), including stroke (OR: 0.96 per SD increment of cystatin C; 95% CI = 0.93–1.01), MI (OR: 0.97 per SD increment of cystatin C; 95% CI = 0.91–1.03), and CVD mortality (OR: 0.98 per SD increment of cystatin C; 95% CI = 0.96–1.01) (Figure 3). The results of linear MR were consistent with the findings concerning the association between cystatin C–GRS and CVDs.

### 3.5. Sensitivity analyses

A series of sensitivity analyses (IVW method, weighted median method, and Mendelian randomization-Egger method) were performed to assess the association between plasma cystatin C and CVDs (Supplementary Figures S2–S5). MR-PRESSO analyses were also conducted to identify potentially pleiotropic outliers. Supplementary Table S6 shows the MR estimates for the association between plasma cystatin C and CVDs. No causal relation was found between plasma cystatin C concentrations and CVDs (IVW method-CVDs: OR = 0.97, 95% CI: 0.84–1.12, *p* = 0.68; stroke: OR = 0.98, 95% CI: 0.78–1.22, *p* = 0.83; MI: OR = 0.95, 95% CI: 0.80–1.13, *p* = 0.58; CVD mortality: OR = 0.70, 95% CI: 0.64–1.36,*p* = 0.56). When the liberal set of 67 cystatin C-associated SNPs was used as instrumental variables, the results were consistent among the four MR methods. The MR-Egger method showed no evidence of directional pleiotropy regarding the association between cystatin C and CVDs (intercept = 0.0005, *p* = 0.91), stroke (intercept = 0.0054, *p* = 0.43), MI (intercept = −0.0014, *p* = 0.79), and CVD mortality (intercept = −0.0024, *p* = 0.99) (Supplementary Table S7). The results of the MR-PRESSO global test showed no potential horizontal pleiotropy (*p* > 0.05).

The results were similar even after further exclusion of SNPs related to confounders and potentially pleiotropic SNPs. Similar results were observed using a set of 15 plasma cystatin C-associated SNPs as instrumental variables (set 1) (Supplementary Table S6).

## 4. Discussion

### 4.1. Key finding

In this large-scale prospective cohort study, observational analysis found that each increase in plasma cystatin C concentration was associated with 9% higher risks of CVD events, 14% higher risks of CVD mortality, 10% higher risks of stroke, and 8% higher risks of MI. In participants with intermediate cystatin C-GRS, elevated plasma cystatin C concentrations were associated with an increased risk of cardiovascular disease and myocardial infarction. Nevertheless, MR estimates showed no significant correlations between plasma cystatin C concentrations and cardiovascular risk. However, recently published MR studies reported controversial results on the association between plasma cystatin C concentrations and CVDs. A 16-cohort MR study showed that decreased cystatin C concentrations were strongly associated with the rs91119 allele, which explained 2.8% of the difference in observational results. However, there was no evidence of a causal relationship between cystatin C concentrations and CVD events in the study (10). The results of our MR study including 277,057 participants with no history of cardiovascular events and cancer did not indicate an effect of plasma cystatin C concentration on cardiovascular risk.

Many previous observational studies have shown that higher cystatin C levels were associated with a higher risk of CVD events and mortality. A community-based longitudinal study based on the Cardiovascular Health Study found that the HR for CVD mortality in the highest cystatin C group (≥1.29 mg/L) was 2.27 (1.73–1.97) compared with the lower group (≤0.99 mg/L), and the HR for MI was 1.48 (1.08–2.02), and the HR for stroke was 1.47 (1.09–1.96). Multivariate modeling adjusted for confounding factors such as age, sex, race, alcohol consumption, BMI, and hypertension (6). Furthermore, meta-analysis showed that serum cystatin levels were significantly associated with the risk of all-cause mortality in the population and suggested that cystatin C levels could be an independent risk factor for CHD (29).

The mechanism of action of cystatin C on CVDs has not been firmly established. When the balance of cystatin C concentrations is disrupted, vascular damage is caused. When coronary arteries become inflamed, inflammatory mediators stimulate vascular smooth muscle to secrete large amounts of cathepsin K and S. Cathepsin promotes the decomposition of collagen and elastic fibers, whereas cystatin C inhibits the activity of cathepsin. This affects the balance of cystatin C concentrations in the body. When cathepsin and cysteine are damaged, their protease activities are enhanced, resulting in vascular tissue damage and vascular wall remodeling (30). After that, cystatin C concentration increased compensatively. Cystatin C can also accelerate the development of atherosclerosis by regulating the activity of cysteine protein kinase to balance the production and degradation of the extracellular matrix. Furthermore, the degradation of the extracellular matrix directly aggravates the rupture of the fibrous cap in coronary atherosclerosis (31). Increases in cystatin C concentrations directly damage endothelial cells, thereby reducing nitric oxide production, altering coagulation factor function, promoting platelet adhesion and aggregation, and causing thrombosis, thereby participating in the occurrence and development of atherosclerosis (32).

Our MR estimates showed no significant correlations between plasma cystatin C and CVDs. We propose several reasons for the inconsistency between our observational and Mendelian findings. First, cystatin C is statistically independent of cardiovascular risk factors, probably because higher cystatin C concentrations reflect the duration and severity of other established risk factors as well, and are associated with long-term exposure to cardiovascular risk (6). Epidemiological studies have demonstrated that cystatin C was independently associated with cardiovascular risk factors (e.g., age, female sex, body mass index, low concentrations of HDL cholesterol, and smoking) (9, 33). In addition, renal dysfunction is associated with cardiac problems, which is linked to CVDs, and cystatin C concentrations are considered markers of renal function. Second, the adverse effects of higher concentrations of cystatin C in patients with CVDs cannot be completely attributed to renal dysfunction. Large cohort studies have reported that higher concentrations of cystatin C and C-reactive protein are significantly associated with a higher risk of CVD events and premature death among the elderly population (34, 35). However, the significant association between cystatin C and C-reactive protein (a marker for inflammation) does not imply a causal relationship, as inflammation plays an important role in the early stages of kidney diseases (33). Third, it has been reported that Cystatin C affects vascular structure by inhibiting cathepsins to reduce matrix degradation (36). Elevated cystatin C concentrations may inhibit ongoing disease processes via compensatory increases in its production.

Recent studies have shown that Cystatin C is not only closely related to the development of CVDs, but also closely related to the prognosis of CVDs patients, and has a certain predictive ability. Correa et al. (37) found that Cystatin C was associated with the prognosis of patients with AMI, suggesting that the higher the Cystatin C level, the worse the prognosis of patients with AMI after percutaneous coronary intervention. A cohort study found that every SD increase in plasma cystatin C was found to be related to 22% higher risks of CVD mortality, 15% higher risks of all-cause mortality, and 27% higher risks of heart failure (38). Similarly, the dose-response relationship between cystatin C levels and the risk of CVD death showed that each 0.1 mg/L increase in cystatin C increased the risk of CVD death by 7.3% (39). These studies suggest that cystatin C may serve as an independent predictor of the risk of developing CVDs.

Our study employed a large sample size and followed a prospective design, which provided adequate outcome events and ensured the large number of covariates on sociodemographic confounders, biological indicators, and more genetic variants, and allowed rigorous adjustment for confounders. Compared with traditional observational studies, this study used MR design to assess the causal association between cystatin C and CVDs in a large prospective cohort. The MR study design can minimize potential biases caused by confounding factors and reverse causality (13). As far as we know, our design was the first genetic analysis of the correlations between plasma cystatin C concentrations and CVDs using linear MR analysis. We implemented several strategies to examine the robustness of our results. First, to minimize the effects of potential confounders or the indications of known pleiotropic effects, we generated two sets of instrumental variables for MR analysis. Second, we examined the relationship between GRS and potential confounders. Third, we conducted the MR-PRESSO global test, leave-one-out analysis, and Cochran’s Q test to assess heterogeneity and the potential horizontal pleiotropy of the genetic variants.

### 4.2. Strengths and limitations

Our study has some limitations. First, single-sample MR studies was likely influenced by weak instrument bias. To tackle this, we calculated the *F*-statistic. Second, our sample only included White British participants. This limits the extrapolation of the study results to other ethnicities, although it minimizes bias in the results obtained by population stratification. Third, although we carefully controlled a number of potential confounders, including demographic factors, lifestyle habits, and kidney function, there remains a chance of residual confounding. Finally, our study included healthy individuals aged 40–70 years at baseline; similar studies conducted with patients in other age groups might be needed to verify our findings in different cohorts.

## 5. Conclusion

In summary, our large and prospective cohort MR study indicates that genetically predicted plasma cystatin C concentrations was not associated to the risk of CVDs and CVD mortality. This suggests that there was no any support for associations between plasma cystatin C and the CVD events and CVD mortality. In our adjusted multivariate observational analysis, higher concentrations of plasma cystatin had increased the risk of total CVD events. Thus, cystatin C is associated with cardiovascular events, but not causally. Our findings do not indicate that cystatin C is an independent risk factor for CVDs; they rather indicate that it is a “marker” for CVDs. Therefore, the detection of serum cystatin C concentrations is helpful for the early diagnosis of CHD, especially for the severity and prognosis of coronary artery disease. Further research on the relationship between cystatin C and CVDs may provide novel insights and prospects for studies into the mechanisms of occurrence and development, diagnosis, treatment, and prognosis evaluation of CHD.

## Data availability statement

The original contributions presented in the study are included in the article/Supplementary material, further inquiries can be directed to the corresponding authors.

## Ethics statement

The studies involving humans were approved by The National Health Service National Research Ethics Service (ref 11/NW/0382). The studies were conducted in accordance with the local legislation and institutional requirements. The participants provided their written informed consent to participate in this study.

## Author contributions

YX and JZ contributed to conception and design of the study. DX and YW organized the database. HS performed the statistical analysis. JT and BZ wrote the first draft of the manuscript. LH, DX, and YW wrote sections of the manuscript. All authors contributed to the article and approved the submitted version.

## Funding

This study is supported by the National Natural Science Foundation of China (82173648), Innovative Talent Support Plan of the Medical and Health Technology Project in Zhejiang Province (2021422878), Internal Fund of Ningbo Institute of Life and Health Industry, University of Chinese Academy of Sciences (2020YJY0212), Ningbo Clinical Research Center for Digestive System Tumors (2019A21003), Ningbo Key Support Medical Discipline (2022-F22), Ningbo Clinical Research Center for Digestive System Tumors (2019A21003), and Sanming Project of Medicine in Shenzhen (SZSM201803080).

## Conflict of interest

The authors declare that the research was conducted in the absence of any commercial or financial relationships that could be construed as a potential conflict of interest.

## Publisher’s note

All claims expressed in this article are solely those of the authors and do not necessarily represent those of their affiliated organizations, or those of the publisher, the editors and the reviewers. Any product that may be evaluated in this article, or claim that may be made by its manufacturer, is not guaranteed or endorsed by the publisher.

## Footnotes

^1^ http://www.ukbiobank.ac.uk

^2^ https://biobank.ndph.ox.ac.uk/showcase/ukb/docs/biomarker_issues.pdf

^3^ https://www.who.int/classifications/icd/en/

^4^ https://github.com/MRCIEU/TwoSampleMR

^5^ https://github.com/rondolab/MR-PRESSO
