# Corrigendum: No causal association between plasma cystatin C and cardiovascular diseases: Mendelian randomization analyses in UK biobank

**DOI:** 10.3389/fmed.2023.1361467

**Published:** 2024-01-08

**Authors:** Jingjing Tu, Ying Xu, Xu Guo, Jiayu Zhang, Duo Xu, Liyuan Han, Yue Wang, Boya Zhang, Hongpeng Sun

**Affiliations:** ^1^Department of Rehabilitation Medicine, Ningbo No.2 Hospital, Ningbo, China; ^2^School of Public Health, Medical College of Soochow University, Suzhou, China; ^3^Department of Global Health, Ningbo Institute of Life and Health Industry, University of Chinese Academy of Sciences, Ningbo, Zhejiang, China

**Keywords:** plasma cystatin C, cardiovascular disease, stroke, myocardial infarction, Mendelian randomization, genetic risk

In the published article, there was an error in the author list. All authors were labeled as first authors. The corrected author list appears below.

“Jingjing Tu^1^, Ying Xu^2^, Xu Guo^1^, Jiayu Zhang^2^, Duo Xu^3^, Liyuan Han^3^, Yue Wang^3^, Boya Zhang^2*^ and Hongpeng Sun^3*^”

The authors apologize for this error and state that this does not change the scientific conclusions of the article in any way. The original article has been updated.

