# Supplementary material for: No causal association between plasma cystatin C and cardiovascular diseases: Mendelian randomization analyses in UK biobank
Source: Front Med (Lausanne). 2023 Aug 17;10:1191675. doi: 10.3389/fmed.2023.1191675 (PMC10472449; doi:10.3389/fmed.2023.1191675)
Supplement: Supplementary file 1 [file Data_Sheet_1.docx]

Supplementary Material

Association between plasma cystatin C and cardiovascular diseases: Mendelian randomization analyses in UK Biobank

Jingjing Tu, Ying Xu, Xu Guo, Jiayu Zhang, Duo Xu, Liyuan Han, Yue Wang, Boya Zhang^*^, Hongpeng Sun^*^

*** Correspondence:**Boya Zhang, MD

E-mail: zhangboya1008@163.com

Hongpeng Sun, PhD

E-mail: hpsun@suda.edu.cn

# Supplementary Figures and Tables

## Supplementary Figures


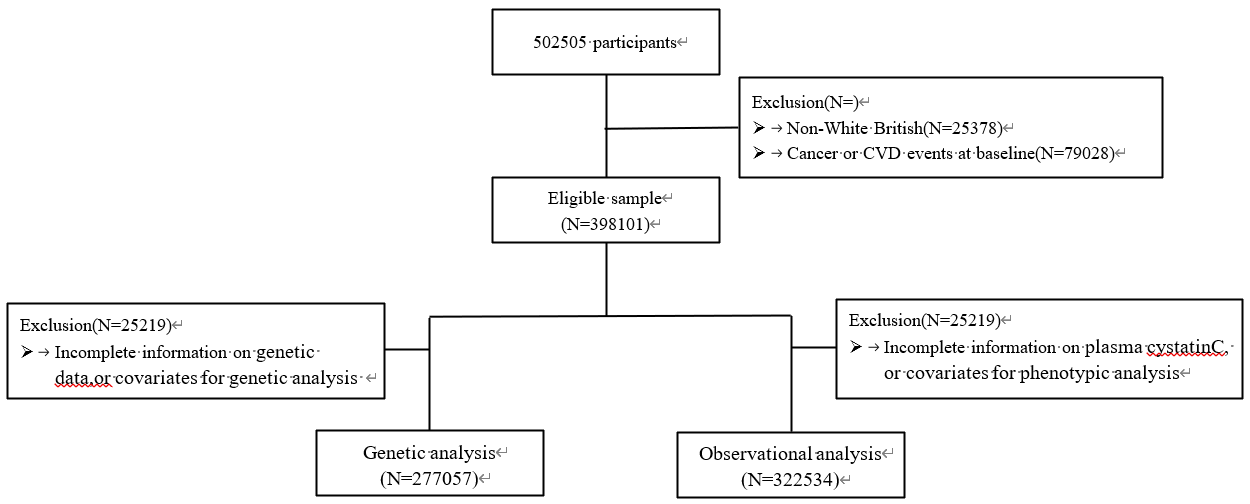


**Supplementary Figure 1.** The flowchart of the UK Biobank study participants


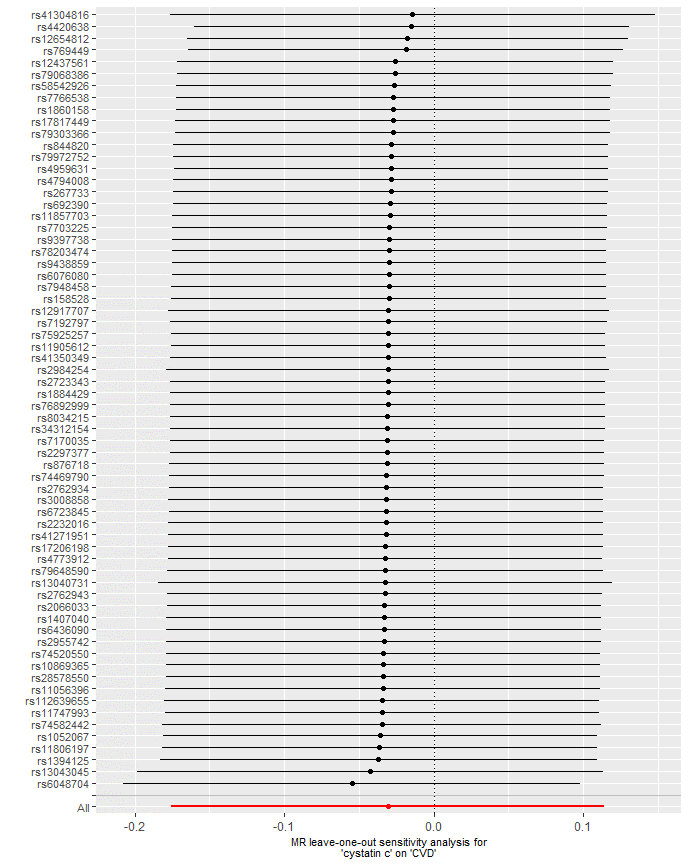


**Supplementary Figure 2.** Forest plot of Mendelian randomization estimates of plasma cystatin C on CVD from each single nucleotide polymorphism


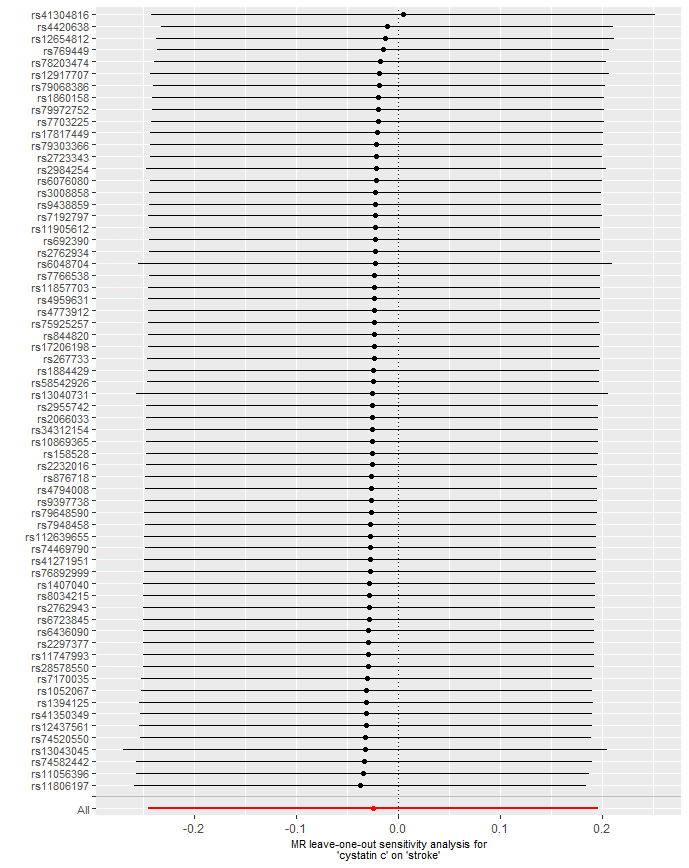


**Supplementary Figure 3.** Forest plot of Mendelian randomization estimates of plasma cystatin C on stroke from each single nucleotide polymorphism


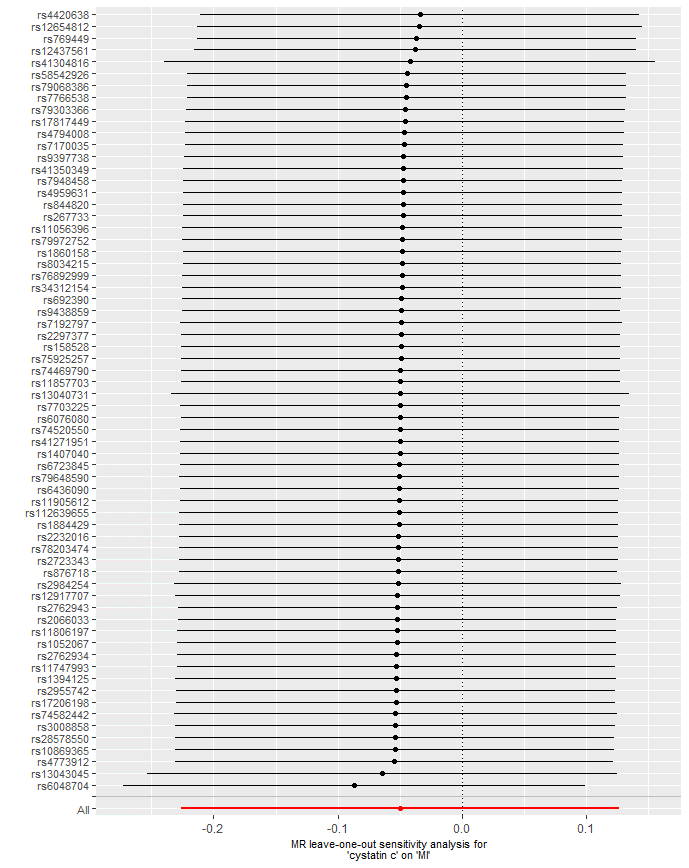


**Supplementary Figure 4.** Forest plot of Mendelian randomization estimates of plasma cystatin C on myocardial infarction (MI) from each single nucleotide polymorphism


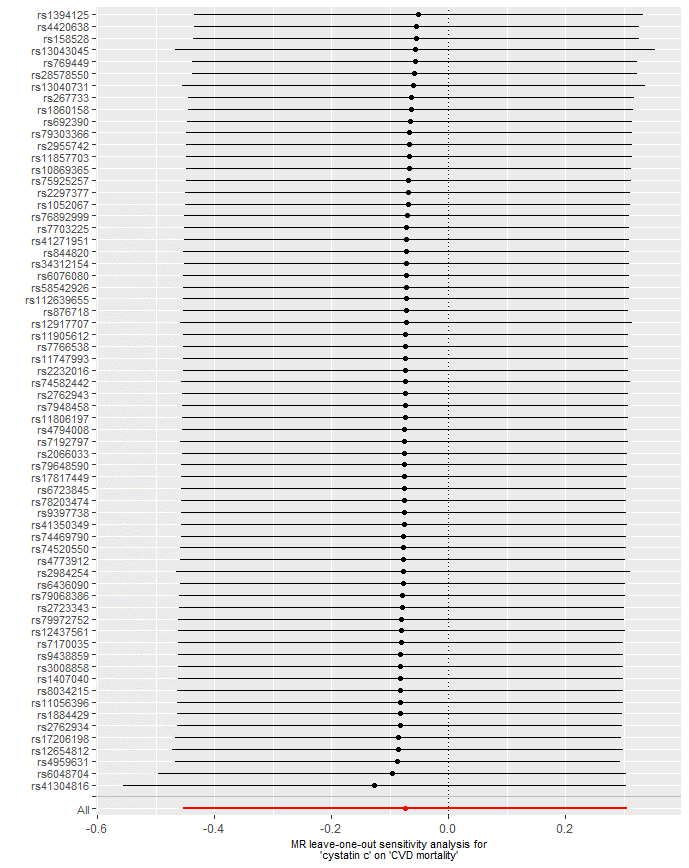


**Supplementary Figure 5.** Forest plot of Mendelian randomization estimates of plasma cystatin C on CVD mortality from each single nucleotide polymorphism

**1.2 Supplementary tables**

**Supplemental table 1.** 67 single-nucleotide polymorphisms and effect sizes for plasma cystatin C identified in UK Biobank.

| SNP | **Chr** | Bp | Gene | Effect | SE | EA | NEA | EAF |
| --- | --- | --- | --- | --- | --- | --- | --- | --- |
| rs41271951 | 1 | 150764744 | CTSS | -0.0291 | 4.34E-03 | A | G | 0.077 |
| rs1884429 | 1 | 11052779 | SRM, MASP2 | -0.0184 | 2.96E-03 | T | C | 0.171 |
| rs9438859 | 1 | 24897830 | CLIC4, RUNX3 | -0.0178 | 2.80E-03 | T | C | 0.266 |
| rs75925257 | 1 | 155166366 | HMGN2P18, KRTCAP2 | 0.0233 | 3.76E-03 | G | A | 0.11 |
| rs1052067 | 1 | 156236330 | PMF1, PMF1-BGLAP | -0.0166 | 2.65E-03 | G | A | 0.277 |
| rs3008858 | 1 | 66924733 | DNAI4 | 0.0233 | 2.71E-03 | C | G | 0.267 |
| rs267733 | 1 | 150986360 | ANXA9 | -0.0281 | 3.26E-03 | A | G | 0.0635 |
| rs2232016 | 1 | 107057296 | PRMT6 | 0.0201 | 2.90E-03 | C | T | 0.192 |
| rs74520550 | 1 | 155033974 | DCST1 | 0.0206 | 2.97E-03 | C | A | 0.199 |
| rs11806197 | 1 | 77499170 | AK5 | 0.0233 | 3.05E-03 | A | G | 0.171 |
| rs78203474 | 2 | 202593004 | BMPR2, MTCO1P17 | 0.0324 | 4.83E-03 | T | C | 0.075 |
| rs876718 | 2 | 15668811 | LINC01804, DDX1 | -0.0247 | 3.95E-03 | G | A | 0.094 |
| rs13419481 | 2 | 202096124 | KIAA2012-AS1, KIAA2012 | 0.0209 | 2.41E-03 | T | A | 0.426 |
| rs6723845 | 2 | 217111868 | intergenic | -0.0237 | 3.40E-03 | T | C | 0.164 |
| rs6436090 | 2 | 218804279 | CYP27A1 | -0.0178 | 2.38E-03 | A | G | 0.493 |
| rs11747993 | 5 | 1193915 | SLC6A19, CTD-3080P12.3 | 0.0168 | 2.73E-03 | G | A | 0.267 |
| rs7703225 | 5 | 177103643 | FGFR4, NSD1 | 0.0288 | 3.47E-03 | G | A | 0.165 |
| rs12654812 | 5 | 177367190 | RGS14 | 0.0449 | 2.50E-03 | G | A | 0.349 |
| rs7766538 | 6 | 2522961 | GMDS-DT | -0.0196 | 2.87E-03 | G | T | 0.218 |
| rs4959631 | 6 | 2097452 | GMDS | -0.0155 | 2.38E-03 | T | C | 0.433 |
| rs9397738 | 6 | 154665530 | SCAF8, RPS4XP8 | -0.0214 | 3.42E-03 | A | G | 0.152 |
| rs1860158 | 7 | 156634322 | LINC01006 | 0.0184 | 2.62E-03 | T | C | 0.298 |
| rs10869365 | 9 | 68824433 | PIP5K1B | -0.0206 | 2.61E-03 | C | T | 0.286 |
| rs74469790 | 10 | 1183975 | LINC00200, ADARB2 | 0.0348 | 4.85E-03 | G | A | 0.068 |
| rs34312154 | 11 | 47448793 | RAPSN | -0.0238 | 3.87E-03 | G | A | 0.116 |
| rs7948458 | 11 | 2151600 | INS-IGF2 | 0.0245 | 3.01E-03 | C | A | 0.23 |
| rs11056396 | 12 | 15191473 | RERG | -0.0309 | 3.60E-03 | T | C | 0.106 |
| rs112639655 | 12 | 57317546 | R3HDM2 | -0.035 | 4.36E-03 | G | A | 0.062 |
| rs79648590 | 12 | 111759793 | ALDH2, ACAD10 | -0.0474 | 4.67E-03 | G | A | 0.081 |
| rs4773912 | 13 | 195504862 | CLDN10, CLDN10-AS1 | -0.0147 | 2.38E-03 | C | T | 0.46 |
| rs2066033 | 13 | 95442527 | CLDN10 | 0.0174 | 2.42E-03 | T | C | 0.451 |
| rs8034215 | 15 | 57441816 | CGNL1 | -0.0183 | 2.68E-03 | A | G | 0.281 |
| rs28578550 | 15 | 41500460 | ITPKA | 0.0191 | 2.87E-03 | G | A | 0.1342 |
| rs7170035 | 15 | 98688639 | IGF1R | -0.0186 | 2.85E-03 | A | G | 0.182 |
| rs692390 | 15 | 41194792 | EXD1 | -0.0199 | 3.08E-03 | G | A | 0.199 |
| rs2297377 | 15 | 41523451 | RPAP1 | -0.0153 | 2.37E-03 | A | G | 0.464 |
| rs11857703 | 15 | 63087393 | LACTB, TPM1 | -0.0221 | 2.50E-03 | G | A | 0.489 |
| rs2723343 | 15 | 71815928 | NR2E3 | -0.023 | 2.95E-03 | C | T | 0.18 |
| rs79972752 | 15 | 75825793 | UBE2Q2, DNM1P49 | -0.0391 | 4.34E-03 | C | T | 0.082 |
| rs12437561 | 15 | 98771392 | IGF1R | 0.0396 | 3.35E-03 | C | T | 0.166 |
| rs41350349 | 15 | 98742151 | IGF1R | 0.0304 | 3.02E-03 | G | A | 0.191 |
| rs1394125 | 15 | 75866642 | UBE2Q2 | 0.0319 | 2.48E-03 | G | A | 0.342 |
| rs2955742 | 15 | 6153791 | TMEM266 | 0.0237 | 3.18E-03 | G | A | 0.144 |
| rs17817449 | 16 | 53779455 | FTO | 0.0222 | 2.43E-03 | T | G | 0.415 |
| rs12917707 | 16 | 20356368 | PDILT, UMOD | -0.0606 | 3.12E-03 | G | T | 0.201 |
| rs7192797 | 16 | 20400441 | PDILT | -0.0358 | 2.64E-03 | G | A | 0.309 |
| rs76892999 | 17 | 48541621 | HOXB2 | -0.0245 | 3.85E-03 | G | A | 0.1 |
| rs4794008 | 17 | 48971599 | GIP, IGF2BP1 | -0.0171 | 2.64E-03 | C | T | 0.268 |
| rs17206198 | 19 | 32824007 | TDRD12 | -0.0233 | 3.47E-03 | A | G | 0.141 |
| rs58542926 | 19 | 19268740 | TM6SF2 | 0.0281 | 4.49E-03 | C | T | 0.068 |
| rs769449 | 19 | 44906745 | APOE | -0.0313 | 3.63E-03 | G | A | 0.123 |
| rs4420638 | 19 | 44919689 | APOC1, APOC1P1 | -0.0296 | 3.04E-03 | A | G | 0.198 |
| rs6076080 | 20 | 23511141 | CST13P, CST8 | 0.0248 | 3.73E-03 | C | T | 0.108 |
| rs11905612 | 20 | 24071013 | LINC01721 | 0.0262 | 3.94E-03 | G | A | 0.098 |
| rs2762934 | 20 | 54154722 | CYP24A1 | -0.0201 | 3.10E-03 | G | A | 0.223 |
| rs1407040 | 20 | 58897119 | GNAS | -0.0168 | 2.51E-03 | T | C | 0.337 |
| rs41304816 | 20 | 23536923 | CST13P | -0.1834 | 9.54E-03 | G | C | 0.099 |
| rs158528 | 20 | 54105417 | CYP24A1, BCAS1 | 0.0228 | 2.53E-03 | G | A | 0.358 |
| rs79068386 | 20 | 24113634 | LINC01721 | 0.0414 | 4.78E-03 | G | A | 0.063 |
| rs79303366 | 20 | 24421086 | GAPDHP53 | 0.0341 | 4.11E-03 | G | A | 0.084 |
| rs2984254 | 20 | 23972468 | CSTP1, GGTLC1 | -0.1048 | 4.96E-03 | C | T | 0.069 |
| rs74582442 | 20 | 23121088 | CD93, LINC00656 | -0.0607 | 4.01E-03 | A | G | 0.083 |
| rs2762943 | 20 | 54174247 | PFDN4, CYP24A1 | 0.0326 | 4.53E-03 | G | T | 0.085 |
| rs13043045 | 20 | 23769767 | CST1, CSTP2 | 0.1598 | 4.26E-03 | C | T | 0.094 |
| rs6048704 | 20 | 23315572 | NXT1-AS1 | -0.0876 | 2.75E-03 | C | T | 0.25 |
| rs13040731 | 20 | 23661752 | CST3, CST4 | -0.1103 | 3.63E-03 | G | A | 0.131 |
| rs844820 | 20 | 23101471 | LINC00656, CD93 | 0.0308 | 3.97E-03 | G | A | 0.107 |

SNP: single-nucleotide polymorphisms; SE: standard error; EA: effect allele; NEA: non-effect allele; EAF: effect allele frequency; CHR: chromosome number;

BP: base-pair position,

**Supplemental table 2.** Classification of SNP function based on GWAS-Catalog, PheWAS

| SNP | Potential pleiotropic effect identified from GWAS catalog and PheWAS | set 1 |
| --- | --- | --- |
| rs41271951 | Calcium, HDL, Glycated haemoglobin, Appendicular lean mass, Heel bone mineral density (BMD) T-score, automated |  |
| rs1884429 | Body mass index, Fat-free mass |  |
| rs9438859 | lymphocyte cell count, Lymphocyte percentage, Low density lipoprotein cholesterol levels |  |
| rs75925257 | Urate, Body mass index (BMI) |  |
| rs1052067 | Urea, Stroke, C-Reactive protein level |  |
| rs3008858 | Lymphocyte counts, Urea |  |
| rs267733 | LDL cholesterol levels, Apolipoprotein A1 levels |  |
| rs2232016 | Total bilirubin levels, Gamma glutamyl transferase levels, Whole body fat mass |  |
| rs74520550 | Urate levels |  |
| rs11806197 | Whole body fat-free mass, Basal metabolic rate |  |
| rs78203474 | / | **√** |
| rs876718 | Serum creatinine (eGFRcrea) |  |
| rs13419481 | / | **√** |
| rs6723845 | Urate levels |  |
| rs6436090 | Triglycerides, Body mass index (BMI) |  |
| rs11747993 | Creatinine levels |  |
| rs7703225 | Urea levels, Creatinine levels |  |
| rs12654812 | Kidney stones, Inflammatory bowel disease, Calcium levels |  |
| rs7766538 | / | **√** |
| rs4959631 | Urate levels |  |
| rs9397738 | Serum creatinine levels, Estimated glomerular filtration rate |  |
| rs1860158 | Haematocrit percentage, Urate, Serum creatinine |  |
| rs10869365 | Serum creatinine levels, Estimated glomerular filtration rate |  |
| rs74469790 | Serum creatinine levels, Estimated glomerular filtration rate |  |
| rs34312154 | Waist-to-hip ratio adjusted for BMI, Apolipoprotein A1 levels, C-reactive protein levels |  |
| rs7948458 | Anthropometric traits, Estimated glomerular filtration rate |  |
| rs11056396 | Gamma glutamyl transferase levels, Urate levels |  |
| rs112639655 | Apolipoprotein B levels, Triglyceride levels |  |
| rs79648590 | / | **√** |
| rs4773912 | Red blood cell (erythrocyte) count, White blood cell count, Urea |  |
| rs2066033 | / | **√** |
| rs8034215 | Creatinine |  |
| rs28578550 | / | **√** |
| rs7170035 | Urate levels |  |
| rs692390 | / | **√** |
| rs2297377 | Apoliprotein A, HDL |  |
| rs11857703 | High density lipoprotein cholesterol levels, Apoliprotein A |  |
| rs2723343 | Urate levels, Haematocrit percentage, Red blood cell (erythrocyte) count |  |
| rs79972752 | Estimated glomerular filtration rate, Urate levels,Urea levels |  |
| rs12437561 | Estimated glomerular filtration rate, Urate levels, Urea levels |  |
| rs41350349 | Urate levels, Urea levels |  |
| rs1394125 | Estimated glomerular filtration rate, Urate levels, Urea levels |  |
| rs2955742 | Estimated glomerular filtration rate, Urate levels, Urea levels |  |
| rs17817449 | Breast cancer, Relative carbohydrate intake |  |
| rs12917707 | Renal function and chronic kidney disease, Kidney function decline traits |  |
| rs7192797 | Estimated glomerular filtration rate, Urate levels, Urea levels, Urate levels |  |
| rs76892999 | / | **√** |
| rs4794008 | / | **√** |
| rs17206198 | Creatinine levels, Estimated glomerular filtration rate |  |
| rs58542926 | Triglycerides, Low density lipoprotein cholesterol levels |  |
| rs769449 | Cingulate cortical amyloid beta load, Cerebrospinal fluid AB1-42 levels |  |
| rs4420638 | total cholesterol measurement, hematocrit, stroke, ventricular rate measurement, body mass index, atrial fibrillation, high density lipoprotein |  |
| rs6076080 | / | **√** |
| rs11905612 | / | **√** |
| rs2762934 | Calcium levels, Vitamin D levels |  |
| rs1407040 | Estimated glomerular filtration rate, Diastolic blood pressure |  |
| rs41304816 | / | **√** |
| rs158528 | Calcium levels, Serum phosphate levels, Estimated glomerular filtration rate |  |
| rs79068386 | / | **√** |
| rs79303366 | Vigorous physical activity, |  |
| rs2984254 | / | **√** |
| rs74582442 | / | **√** |
| rs2762943 | Serum 25-Hydroxyvitamin D levels, Estimated glomerular filtration rate, Vitamin D levels |  |
| rs13043045 | / | √ |
| rs6048704 | / | √ |
| rs13040731 | / | √ |
| rs844820 | cytokine measurement | √ |

*Set 1: SNP excluded if associated with potential causes of confounders based on public datasources (PheWAS and GWAS Catalog) and UK Biobank

**Supplemental table 3**. Association of cystatin C -GRS with potential confounders in the UK Biobank.

|  |  | **Cystatin C -GRS** | P |
| --- | --- | --- | --- |
|  | N (%) | Mean (S.D.) |  |
| BMI |  |  |  |
| <25 kg/m^2^ | 96462(34.82) | -0.01(0.02) | 0.0874 |
| (25,30) kg/m^2^ | 117684(42.48) | -0.01(0.02) |  |
| ≥30 kg/m^2^ | 62911(22.71) | -0.01(0.02) |  |
| Smoking |  |  |  |
| Never-smoker | 152554(55.27) | -0.01(0.02) | 0.5923 |
| Former smoker | 97694(35.39) | -0.01(0.02) |  |
| Current smoker | 25778(9.34) | -0.01(0.02) |  |
| Drinking status |  |  |  |
| Never-drinker | 8311(3) | -0.02(0.02) | 0.9231 |
| Former drinker | 8549(3.09) | -0.01(0.02) |  |
| Current drinker | 259965(93.91) | -0.01(0.02) |  |
| Physical activity(min/week) |  |  |  |
| <250 | 123231(55.68) | -0.01(0.02) | 0.9397 |
| 250-550 | 52828(23.87) | -0.01(0.02) |  |
| >550 | 45278(20.46) | -0.01(0.02) |  |
| Income |  |  |  |
| <18000 | 48842(20.4) | -0.01(0.02) | 0.4972 |
| 18000-52000 | 123900(51.74) | -0.01(0.02) |  |
| 52000-100000 | 52267(21.83) | -0.01(0.02) |  |
| >100000 | 14469(6.04) |  |  |
| Townsend deprivation  Index quartiles |  |  |  |
| <0.47 | 196595(70.96) | -0.01(0.02) | 0.1227 |
| ≥0.47 | 80462(29.04) | -0.01(0.02) |  |
| HDL-C (mmol/L) |  |  | 0.8289 |
| <1.4 | 145500(52.52) | -0.01(0.02) |  |
| >1.4 | 131557(47.48) | -0.01(0.02) |  |
| LDL-C (mmol/L) |  |  |  |
| <3.7 | 159405(57.54) | -0.01(0.02) | 0.0108 |
| ≥3.7 | 117652(42.46) | -0.02(0.02) |  |
| Triglyceride (mmol/L) |  |  |  |
| <1.69 | 168147(60.69) | -0.01(0.02) | 0.7737 |
| ≥1.69 | 108910(39.31) | -0.01(0.02) |  |
| Cholesterol (mmol/L) |  |  |  |
| <5.89 | 158614(57.25) | -0.01(0.02) | 0.1609 |
| ≥5.89 | 118443(42.75) | -0.02(0.02) |  |

P values have been adjusted for age, sex, genotyping array

**Supplemental table 4.** Summary characteristics of UK Biobank participants in datasets used for analyses

| **Variable** | **Observational analysis** | **Mendelian randomization** |
| --- | --- | --- |
| N | 372882 | 277057 |
| Age (years) | 56.92(8.07) | 56.90(8.08) |
| Sex, male (%) | 167980(45.05%) | 123526(44.59%) |
| BMI (kg/m2) | 27.27(4.70) | 27.21(4.66) |
| Townsend deprivation index | -1.51(2.96) | -1.56(2.93) |
| Smoking status |  |  |
| Never-smoker | 203719(54.63%) | 152554(56.06%) |
| Former smoker | 129703(34.78%) | 97694(35.26%) |
| Current smoker | 38227(10.25%) | 25778(9.30%) |
| No response | 1233(0.33%) | 1031(0.37%) |
| Drinking status |  |  |
| Never-drinker | 11390(3.05%) | 8311(3.00%) |
| Former drinker | 11901(3.19%) | 8549(3.09%) |
| Current drinker | 349285(93.67%) | 259965(93.83%) |
| No response | 306(0.08%) | 232(0.08%) |
| Employment |  |  |
| no | 150484(40.36%) | 111566(40.27%) |
| yes | 219475(58.86%) | 163309(58.94%) |
| No response | 2923(0.78%) | 2182(0.79%) |
| Physical activity(min/week) |  |  |
| <250 | 165063(55.77%) | 123231(55.68%) |
| 250-550 | 70848(23.79%) | 52828(23.87%) |
| >550 | 61266(20.44%) | 45278(20.46%) |
| Income |  |  |
| <18000 | 67248(21.39%) | 48842(20.40%) |
| 18000-52000 | 166802(51.51%) | 123900(51.74%) |
| 52000-100000 | 69514(21.28%) | 52267(21.83%) |
| >100000 | 18970(5.82%) | 14469(6.04%) |
| HDL, mmol/L | 1.45(0.37) | 1.46(0.37) |
| LDL, mmol/L | 3.57(0.86) | 3.58(0.86) |
| Cholesterol mmol/L | 5.73(1.14) | 5.73(1.13) |
| Triglycerides ,mmol/L | 1.73(1.01) | 1.72(1.00) |
| Glucose,mmol/L | 5.08(1.13) | 5.08(1.11) |
| Cystatin C,mg/L | 0.90(0.16) | 0.90(0.16) |
| Creatinine,umol/L | 71.96(16.52) | 71.89(16.42) |
| Diabetes | 15925(4.27%) | 11416(4.12%) |
| Hypertension | 143757(38.55%) | 105978(38.25%) |
| CKD | 8932(2.40%) | 6706(2.42%) |

Values were expressed as mean ± standard deviation, or n (%).

CKD: chronic kidney disease

**Supplemental table 5.** Adjusted hazard ratios and 95% confidence intervals for serum Cystatin C with the risk of CVD events and CVD mortality in UK Biobank study

|  | Hazard ratio (95% confidence interval) | | | | | | *P*-trend |
| --- | --- | --- | --- | --- | --- | --- | --- |
|  | Quintiles 1 | Quintiles 2 | Quintiles 3 | Quintiles 4 | Quintiles 5 | For each standard deviation increase |  |
| Cystatin C(median ,mg/L) | 0.74 | 0.82 | 0.88 | 0.95 | 1.07 |  |  |
| CVD events |  |  |  |  |  |  |  |
| No. of cases | 1775 | 2680 | 3491 | 4755 | 8802 |  |  |
| model 1 | 1.0(ref) | 1.07(0.98,1.77) | 1.13(1.04,1.24) | 1.28(1.18,1.39) | 1.74(1.60,1.88) | 1.14(1.13,1.15) | <.0001 |
| model 2 | 1.0(ref) | 1.10(1.00,1.20) | 1.14(1.05,1.25) | 1.26(1.16,1.37) | 1.55(1.43,1.69) | 1.11(1.09,1.12) | <.0001 |
| model 3 | 1.0(ref) | 1.09(1.00,1.20) | 1.14(1.04,1.24) | 1.25(1.16,1.15) | 1.50(1.38,1.63) | 1.09(1.07,1.10) | <.0001 |
| Stroke |  |  |  |  |  |  |  |
| No. of cases | 862 | 1201 | 1441 | 1821 | 3565 |  |  |
| model 1 | 1.0(ref) | 1.05(0.92,1.21) | 1.07(0.94,1.22) | 1.12(0.98,1.27) | 1.53(1.34,1.73) | 1.14(1.13,1.16) | <.0001 |
| model 2 | 1.0(ref) | 1.06(0.93,1.21) | 1.08(0.95,1.24) | 1.13(0.99,1.29) | 1.60(1.41,1.82) | 1.12(1.10,1.14) | <.0001 |
| model 3 | 1.0(ref) | 1.05(0.92,1.21) | 1.07(0.94,1.22) | 1.17(1.00,1.27) | 1.50(1.33,1.70) | 1.10(1.08,1.11) | <.0001 |
| Myocardial infarction |  |  |  |  |  |  |  |
| No. of cases | 974 | 1575 | 2207 | 3164 | 5897 |  |  |
| model 1 | 1.0(ref) | 1.11(0.98,1.24) | 1.21(1.08,1.36) | 1.39(1.25,1.55) | 1.85(1.66,2.05) | 1.14(1.13,1.15) | <.0001 |
| model 2 | 1.0(ref) | 1.12(0.99,1.26) | 1.19(1.06,1.33) | 1.32(1.18,1.47) | 1.56(1.40,1.74) | 1.10(1.09,1.12) | <.0001 |
| model 3 | 1.0(ref) | 1.12(0.99,1.25) | 1.18(1.06,1.32) | 1.31(1.18,1.47) | 1.52(1.36,1.69) | 1.08(1.07,1.10) | <.0001 |
| CVD mortality |  |  |  |  |  |  |  |
| No. of cases | 173 | 284 | 365 | 546 | 1451 |  |  |
| model 1 | 1.0(ref) | 1.04(0.78,1.38) | 1.12(0.86,1.47) | 1.29(1.00,1.68) | 2.02(1.58,2.79) | 1.18(1.15,1.20) | <.0001 |
| model 2 | 1.0(ref) | 1.02(0.77,1.35) | 1.08(0.82,1.41) | 1.20(0.93,1.56) | 1.77(1.37,2.28) | 1.16(1.14,1.19) | <.0001 |
| model 3 | 1.0(ref) | 1.02(0.77,1.35) | 1.08(0.82,1.42) | 1.21(0.93,1.57) | 1.70(1.32,2.16) | 1.14(1.11,1.17) | <.0001 |

Hazard Ratios (HRs) at each category Q2-Q5 (compared with Q1) and per 1-standard deviation (SD) of each plasma cystatin C, estimated from Cox regression models. P value for trend was calculated as the trend per group.

Model 1:adjusted for age, sex, Townsend Deprivation Index(continuous), household income (<￡18 000, ￡18 000-￡52000,￡52000-￡100000, or >￡100000) , physical activity (<250min/week,250-550 min/week,>550min/week), smoking status(never, former, current), drinking status (never, former, current), employment (no, yes). Model 2: model 1 + BMI (continuous), HDL cholesterol (continuous), LDL cholesterol (continuous) and total cholesterol. Model 3: model 2 + diabetes (yes or no), hypertension (yes or no) and chronic kidney disease (yes or no).

**Supplemental table** **6**. Mendelian randomisation analyses of the effect of serum Cystatin C on risk of CVD events and CVD mortality in the UK Biobank

| **Outcomes** | **Instrument** | **MR Methods** | **NO.SNPs** | **Odds ratio** | **95% confidence interval** | **p-value** |
| --- | --- | --- | --- | --- | --- | --- |
| **CVD** | all snps | MR Egger | 66 | 0.96 | (0.77,1.20) | 0.72 |
|  |  | Weighted median | 66 | 0.98 | (0.80,1.20) | 0.86 |
|  |  | Inverse variance weighted | 66 | 0.97 | (0.84,1.12) | 0.68 |
|  |  | MR-PRESSO | 66 | 0.97 | (0.06,1.06) | 0.54 |
|  | set 1 | MR Egger | 19 | 1.04 | (0.80,1.35) | 0.79 |
|  |  | Weighted median | 19 | 0.99 | (0.79,1.23) | 0.92 |
|  |  | Inverse variance weighted | 19 | 0.98 | (0.82,1.17) | 0.79 |
|  |  | MR-PRESSO | 19 | 0.98 | (0.10,1.11) | 0.75 |
| **Stroke** | all snps | MR Egger | 66 | 0.88 | (0.62,1.23) | 0.45 |
|  |  | Weighted median | 66 | 0.96 | (0.69,1.32) | 0.78 |
|  |  | Inverse variance weighted | 66 | 0.98 | (0.78,1.22) | 0.83 |
|  |  | MR-PRESSO | 66 | 0.98 | (0.09,1.09) | 0.67 |
|  | set 1 | MR Egger | 19 | 0.95 | (0.64,1.42) | 0.82 |
|  |  | Weighted median | 19 | 0.96 | (0.68,1.34) | 0.80 |
|  |  | Inverse variance weighted | 19 | 0.93 | (0.71,1.22) | 0.58 |
|  |  | MR-PRESSO | 19 | 0.93 | (0.05,1.05) | 0.24 |
| **Myocardial infarction** | all snps | MR Egger | 66 | 0.98 | (0.74,1.29) | 0.88 |
|  |  | Weighted median | 66 | 0.96 | (0.75,1.23) | 0.76 |
|  |  | Inverse variance weighted | 66 | 0.95 | (0.80,1.13) | 0.58 |
|  |  | MR-PRESSO | 66 | 0.96 | (0.06,1.06) | 0.39 |
|  | set 1 | MR Egger | 19 | 1.06 | (0.77,1.46) | 0.73 |
|  |  | Weighted median | 19 | 0.98 | (0.74,1.30) | 0.90 |
|  |  | Inverse variance weighted | 19 | 0.99 | (0.80,1.23) | 0.94 |
|  |  | MR-PRESSO | 19 | 1.00 | (0.14,1.15) | 0.99 |
| **CVD mortality** | all snps | MR Egger | 66 | 0.92 | (0.54,1.74) | 0.99 |
|  |  | Weighted median | 66 | 0.88 | (0.61,1.78) | 0.98 |
|  |  | Inverse variance weighted | 66 | 0.70 | (0.64,1.36) | 0.56 |
|  |  | MR-PRESSO | 66 | 0.99 | (0.59,1.68) | 0.26 |
|  | set 1 | MR Egger | 19 | 1.16 | (0.59,2.29) | 0.66 |
|  |  | Weighted median | 19 | 1.07 | (0.59,1.93) | 0.83 |
|  |  | Inverse variance weighted | 19 | 0.92 | (0.58,1.47) | 0.74 |
|  |  | MR-PRESSO | 19 | 0.94 | (0.16,1.18) | 0.58 |

SNP, single nucleotide polymorphism;CI: confidence interval; GWAS, genome wide association studies; CVD: cardiovascular disease;

MR-PRESSO, Mendelian randomization pleiotropy residual sum and outlier;

Set 1: SNP excluded if associated with potential causes of blood pressure or confounders based on public datasources (PhenoScanner and GWAS Catalog) and UK Biobank.

**Supplemental table 7.**MR-Egger test of directional pleiotropy

| Outcomes | Instrument | Intercept (95% CI) | P-value |
| --- | --- | --- | --- |
| CVD |  |  |  |
|  | all snp(66) | 0.0005(-0.0083,0.0093) | 0.909 |
|  | set 1(19) | -0.0055(-0.0230,0.0120) | 0.545 |
| Stroke |  |  |  |
|  | all snp(66) | 0.0054(-0.0079,0.0187) | 0.423 |
|  | set 1(19) | -0.0027(-0.0288,0.0235) | 0.844 |
| Myocardial infarction |  |  |  |
|  | all snp(66) | -0.0014(-0.0121,0.0093) | 0.792 |
|  | set 1(19) | -0.006(-0.0275,0.0154) | 0.589 |
| CVD mortality |  |  |  |
|  | all snp(66) | -0.0024(-0.0252,0.0204) | 0.999 |
|  | set 1(19) | -0.0216(-0.0673,0.0241) | 0.368 |

**Supplemental table 8.** Tests of Heterogeneity in the SNP-exposure association

| Outcomes | Instrument | Methods | Q | df | P-value |
| --- | --- | --- | --- | --- | --- |
| CVD | all snp(66) | Inverse-variance weighted | 24.49 | 64 | 0.99 |
|  |  | MR Egger | 24.51 | 65 | 0.99 |
|  | set 1(19) | Inverse-variance weighted | 7.96 | 17 | 0.96 |
|  |  | MR Egger | 8.34 | 18 | 0.97 |
| Stroke | all snp(66) | Inverse-variance weighted | 16.63 | 64 | 0.99 |
|  |  | MR Egger | 17.28 | 65 | 0.99 |
|  | set 1(19) | Inverse-variance weighted | 3.76 | 17 | 0.99 |
|  |  | MR Egger | 3.80 | 18 | 0.99 |
| Myocardial infarction | all snp(66) | Inverse-variance weighted | 20.23 | 64 | 0.99 |
|  |  | MR Egger | 20.30 | 65 | 0.99 |
|  | set 1(19) | Inverse-variance weighted | 7.43 | 17 | 0.97 |
|  |  | MR Egger | 7.73 | 18 | 0.98 |
| CVD mortality | all snp(66) | Inverse-variance weighted | 15.32 | 64 | 0.99 |
|  |  | MR Egger | 15.36 | 65 | 0.99 |
|  | set 1(19) | Inverse-variance weighted | 3.28 | 17 | 0.99 |
|  |  | MR Egger | 4.13 | 18 | 0.99 |
